# Tree-based analysis of longevity predictors and their ten-year changes: a 35-Year mortality follow-up

**DOI:** 10.1186/s12877-024-05404-4

**Published:** 2024-10-11

**Authors:** Lily Nosraty, Jaakko Nevalainen, Jani Raitanen, Linda Enroth

**Affiliations:** 1 Faculty of Social Sciences, Centre of Excellence in Research on Ageing and Care, University of Helsinki, Helsinki, Finland; 2 Faculty of Social Sciences (Health Sciences) and Gerontology Research Center (GEREC), Tampere University, Tampere, Finland; 3 Faculty of Social Sciences (Health Sciences), Tampere University, Tampere, Finland; 4 The UKK Institute for Health Promotion Research, Tampere, Finland

**Keywords:** Mortality, Relative measure of longevity, Machine learning, Regression tree, Realized probability of dying

## Abstract

**Background:**

Prior studies on longevity often examine predictors in isolation and rely solely on baseline information, limiting our understanding of the most important predictors and their dynamic nature. In this study, we used an innovative regression tree model to explore the common characteristics of those who lived longer than their age and sex peers in 35-years follow-up. We identified different pathways leading to a long life, and examined to how changes in characteristics over 10 years (from 1979 to 1989) affect the findings on longevity predictors.

**Methods:**

Data was obtained from the “Tampere Longitudinal Study on Ageing” (TamELSA) in Finland. Survey data was collected in 1979 from 1056 participants aged 60–89 years (49.8% men). In 1989, a second survey was conducted among 432 survivors from the 1979 cohort (40.2% men). Dates of death were provided by the Finnish Population Register until 2015. We employed an individual measure of longevity known as the realized probability of dying (RPD), which was calculated based on each participant’s age and sex, utilizing population life tables. RPD is based on a comparison of the survival time of each individual of a specific age and sex with the survival time of his/her peers in the total population. A regression tree analysis was used to examine individual-based longevity with RPD as an outcome.

**Results:**

This relative measure of longevity (RPD) provided a complex regression tree where the most important characteristics were self-rated health, years of education, history of smoking, and functional ability. We identified several pathways leading to a long life such as individuals with (1) good self-rated health (SRH), short smoking history, and higher education, (2) good SRH, short smoking history, lower education, and excellent mobility, and (3) poor SRH but able to perform less demanding functions, aged 75 or older, willing to do things, and sleeping difficulties. Changes in the characteristics over time did not change the main results.

**Conclusion:**

The simultaneous examination of a broad range of potential predictors revealed that longevity can be achieved under very different conditions and is achieved by heterogeneous groups of people.

**Supplementary Information:**

The online version contains supplementary material available at 10.1186/s12877-024-05404-4.

## Background

Mortality rates have declined, however, the probability of dying increases exponentially with age [1]. As individuals age, variability in health outcomes becomes more pronounced, contributing to a growing heterogeneity among older adults. The growing heterogeneity in health and longevity has been associated with differences in genetics, physiological and psychological function, living environment [2], socio-economic background [3, 4], and the potential of risk due to behaviors and accidents [5].

Studies associate longevity with risk behavior predictors like smoking and physical inactivity [6], psychological predictors such as social relations [7], life satisfaction [8], feeling tired of life [9], loneliness [10], and self-rated health (SRH) [11, 12]. Potential predictors are often studied separately [6], making it hard to identify the most important predictors of longevity and to assess their individual and joint contributions to it. For example, focusing an analysis only on a single predictor that is associated with other predictors can result in an exaggeration of its effect size [13]. Thus, it is important to incorporate predictors across different domains in order to identify the most important predictors of longevity [2].

In general, aging involves health deterioration and a decline in functioning [14]. Previous studies have mainly focused on using baseline predictors of mortality without considering the dynamic nature of the predictors [15] or the emergence of new conditions during follow-up [15]. Changes in functioning may impact other characteristics like self-rated health (SRH) or life satisfaction [16]. By tracking these evolving characteristics over time, longitudinal data provides a unique opportunity to reveal how the aging process unfolds and impacts various aspects of health and well-being.

Older adults are a heterogeneous group, not only in terms of their health, living conditions, and social activities, but also because ‘old age’ encompasses a broad age range, often spanning more than 40 years. This diversity highlights the need for individualized analysis, as significant differences in health trajectories and life circumstances persist within this age group. Previous research on longevity predictors has often used Cox regression analysis for both short and long follow-up times, which may not deeply consider heterogeneity, even with age adjustment [17]. Specifically, the Cox model struggles to capture non-linearities and interactions between multiple predictors, and outputs are built on the assumption of proportional hazards [18]. For example, individuals differ in functioning, health and lifestyle factors, and environmental exposures that collectively influence their potential for longevity. Yet many studies have not fully addressed this multifaceted heterogeneity [17]. Deeg et al. (2018) and Rutherford et al. (2012) proposed that individual-based measures of survival time, particularly for long follow-up time and wide age ranges, can better capture heterogeneity and improve longevity prediction accuracy [19, 20]. Consequently, new approaches are needed to address this issue and to ponder the dynamic nature of the characteristics predicting longevity.

In this study, we use an age- and sex-specific individual-based longevity measure to explore a wide range of longevity predictors among older people. We relate each individual’s longevity to the actuarial life expectancy of their age and sex cohort. For this method to be effective, it is essential to have a sufficiently large and representative sample, a long enough follow-up period during which the majority of participants have died, together with precise survival time data, and a broad set of potential predictors [19]. More specifically, we examine: (1) common characteristics among those reaching longevity in a long follow-up, and (2) to what extent changes in characteristics over the years affect the findings. We examine older adults aged 60–89 (at 1979) in a 35-year follow-up, and changes in the longevity predictors between 1979 and 1989. These research questions are explored using an algorithmic tree-based method, which unlike many traditional parametric models, does not impose assumptions about the underlying distribution or form of relationships between variables. However, a regression tree does impose a structure by predicting the mean outcome within each leaf.

## Methods

### Data

Data were obtained from the prospective “Tampere Longitudinal Study on Ageing” (TamELSA) in Finland. Data were collected in 1979 and 1989 using structured questionnaires in face-to-face interviews. The baseline study population in 1979 consisted of 1056 participants aged 60–89 years, of whom 49.8% were men (81% response rate). In 1989, a second measurement round was conducted among 432 survivors from the 1979 cohort (40.2% men, response rate 84%).

The dates of death were provided by the Finnish Population Register. This information was linked to the TamELSA data using personal identification codes. Vital status was ascertained up to the 1st of January 2015. At that time, 0.95% of the participants were still alive.

The research was conducted in accordance with ethical guidelines outlined in the Declaration of Helsinki, and the permission to use national mortality registers was granted by the national register authority, Statistics Finland, and the Population Register.

### Longevity measure: realized probability of dying (RPD)

RPD is a relative measure of longevity that is based on the comparison of the survival time of each individual of a specific age and sex with the survival time of his/her peers in the total population [19, 21]. A life table of the Finnish population was used for obtaining the survival time of peers in the total population [22]. Based on the probability of dying in a given year from the baseline of the study onwards, the RPD was calculated separately for men and women using the formula presented by Deeg et al. [19].

In the formula: RPD = (1 – d_1_^(ai., s)^) x (1 – d_2_^(ai., s)^) x … x (1 – d_n_^(ai., s)^), where n is the total number of calendar years during which the participant is followed up to death or end-of-study, d_n_^(ai, s)^is the probability of death according to the life table in calendar year i (i = 1, 2, …, n) for a participant “a” years of age and “s” of sex" [19, 21].

An individual’s RPD is expressed as the proportion of the pertinent cohort still alive at the time of death of the individual [19, 21]. Values are between 0 and 1, with higher values indicating a shorter survival compared to others of the same age and sex. For example, if 80% of the cohort is still alive at the time of death, then the RPD value of the individual is 0.80.

Less than 1% (*n* = 10) of the study participants were alive at the end of the study. For them, RPD was imputed by multiplying the age and sex-specific probability of survival in 2014 by 0.50.

### Predictors of longevity: individual’s characteristics and their changes

Baseline measures of individual characteristics and changes in characteristics occurring between 1979 and 1989 were considered as potential longevity predictors. *Sociodemographic characteristics* included sex, marital status, years in full-time education, and social class (based on main occupation). *Health and functioning* were addressed as self-reported diseases, SRH, activities of daily living (ADL), mobility activities, demanding functioning (cut toenails, cook, light housework, and heavy housework), number of medications, weight loss, sleeping difficulties, and hearing problems. In addition, health behavior was measured with years of smoking, and physical exercise. *Subjective experiences* were measured with satisfaction from social relations, economic situation and life, and feelings such as feeling of worsening memory, feeling unnecessary, and feeling tired of life. *Social functioning* was addressed with social activities, assisting in bringing-up grandchildren, last paid or received visit, and having good friends. *Living conditions* were measured by having a telephone, fridge, freezer, and using a car. Predictors are described in detail in the appendix.

There were 49 baseline variables and 40 variables with two measurements at different times (described in detail in the appendix). The variables that were subject to change between 1979 and 1989 are highlighted in the table of the appendix. The changes in the scores of variables were quantified as worsened, unchanged, and improved.

### Analysis

As the distribution of the RPD was almost uniform, a logit-transformation of RPD (LRPD) i.e., log (RPD / (1 – RPD)) was used. The log-transformed version was used because this has been used in earlier studies on the same and different data, and in this way our examination is comparable with earlier studies [19, 21].

We used a tree-based model, specifically a regression tree [23] with a sequence of branching or partition operations, in order to identify the predictors of LRPD. The analysis was conducted using two data sets: (1) the baseline data from 1979, and (2) the baseline data together with changes in the variables from 1979 to 1989. To build a single regression tree, recursive partitioning of the data set was used to sequentially split it into non-overlapping subsets of participants, based on their predictor values. At each step, the ‘parent’ node was split by identifying the predictor variable with the best discriminative value and determining the optimal cut point within that variable. The goal was to partition the data so that the responses (LRPD) in the resulting ‘child’ nodes were as similar as possible. This process was continued until the stopping criterion was satisfied for each ‘terminal’ node, which was the default stopping criterion of SAS HPSPLIT. Participants with missing values were assigned to nodes based on observed predictor similarities, using the software’s default. The model performance was assessed using 10-fold cross-validation [23]. The analyses were conducted using SAS Software 9.4 and the HPSPLIT procedure. Tree-based models require the specification of several hyperparameters. For the primary analysis, we used the default values provided by the software procedure. To assess the sensitivity of the results to different hyperparameter settings, we varied the maximum tree depth (maxdepth), minimum leaf size (minleafsize), and minimum variance required for a split (minvariance). The hyperparameters were adjusted to: maxdepth = 10 (default = 5), minleafsize = 10 (default = 1), and minvariance = 0.01 (default = 10^(-8)). Although cross-validation was not explicitly used to tune these hyperparameters in the primary analysis, we conducted a sensitivity analysis to determine how variations in these values impacted the model’s performance.

## Results

The mean values of RPD and LRPD, were 0.47 (standard deviation (SD) 0.28, range 0 to 1), and − 0.22 (SD 1.59, range − 5.6 to 5.0) respectively. The characteristics (mean, median, or percentage) of the predictor variables that were used in building the tree are listed in Table 1 for the cohort of 1979 (*N* = 1056) and for survivors from 1979 to 1989.

**Table 1:** The characteristics (mean, median, or percentage) of the cohort 1979 (N=1056) and of survivors from 1979 to 1989 (N=435)

Domains	Variable	Baseline 1979 (%)	Survivors 1979-89 (%)		Variable	Baseline 1979 (%)	Survivors 1979-89 (%)
Sociodemographic	Gender (male)	50.0	40.5		Having children		
	Age (mean)	74.0	79.2		No	23.1	21.5
	Marital status				Social class (based on occupation) ^a^		
	Married	45.9	37.5		Manual	95.8	87.3
	Never married	13.4	13.0		Non-manual	6.2	12.5
	Widowed	35.4	42.4		Years in full-time education	4.0	5.0
	Divorced	5.4	7.17				
Health, Diseases, and disabilities	5 ADL activities ^b^ (median)	15.0	15.0		Neoplasm	2. 8	5.6
	Able	80.3	71.2		Endocrine diseases	12.6	20.1
	Functional mobility ^d^	14.0	12.0		Diseases of blood	1.7	4.0
	(median)				Mental disorder	3.5	7.7
	Able	47.3	30.4		Nervous system diseases	24.6	30.2
	Demanding functioning ^d^	10.0	9.0		Hypertension	23.1	20.0
	(median)				Cardiac ischemic	13.7	18.7
	Able	40.7	26.0		Circulatory	38.2	38.9
	Self-rated health				Respiratory	12.2	10.5
	Bad	8.0	10.4		Digestive	14.0	16. 8
	Fairly bad	19.2	21.6		Genito-urinary	9.2	8.7
	Average	35.8	34.9		Musculoskeletal	35.6	35.8
	Fairly good	27.9	26.2		Weight lost		
	Good	9.0	6.9		Yes, nearly continuously	0.5	0.5
	Memory worsening				Yes, often	0.8	0.8
	Yes, nearly	18.8	5.9		Yes, occasionally	3.7	4.1
	continuously				No	95.0	94.6
	Yes, occasionally	13.4	14.0		Hearing	52.2	56.0
	Yes, often	27.4	28.3		Physical exercise		
	No	40.5	51.8		Yes	0.5	61.3
	Depressiveness				Years smoked regularly	30.0	30.0
	Yes, nearly continuously	5.6	3.1		(median)		
	Yes, often	5.3	6.7		Number of medications	1.0	2.0
	Yes, occasionally	20.6	26.2		(median)		
	No	68.6	64.1				
Subjective experiences	Pain				Feeling forgotten		
	Yes, nearly continuously	24.8	21.4		Often + cannot say	4.2	4.7
	Yes, often	13.7	15.1		Sometimes	17.8	19.3
	Yes, occasionally	21.6	25.0		Never	78.1	76.0
	No	40.0	38.5				
	Satisfied with present life				Feeling unnecessary		
	Cannot say + very unsatisfied	3.0	3.6		Often	12.3	11.0
	Unsatisfied	3.5	2.8		Sometimes + cannot say	26.2	28.9
	Reasonably satisfied	23.0	19.3		Never	61.5	60.1
	Satisfied	41.9	44.3		Feeling tired of life		
	Very satisfied	28.6	30.0		Often	6.3	6.6
	Satisfaction with the economic situation				Sometimes + cannot say	27.3	28.9
	Bad + cannot say	8.3	5.0		Never	66.4	64.5
	Satisfactory	67.3	60.6		Loneliness		
	Good	24.4	34.4		Cannot say + often	11.1	13.7
	Unwilling to do things or lack of energy				Sometimes	23.9	29.3
	Yes, nearly continuously	18.8	12.5		Never	65.0	57.0
	Yes, often	13.4	11.5		Fatigue & tiredness feeling	54.8	47.1
	Yes, occasionally	27.4	23.0		Satisfied with human relationship		
	No	40.5	53.1		Satisfied	84.7	78.0
					Unsatisfied + cannot say	9.0	6.0
Social Activities	Social activities ^e^	10.0	10.0		Having good friends	80.8	82.9
	Social contacts				Helping children	27.2	84.3
	Last paid visit ^f^ (median)	4.0	4.0		Being alone (yes)	55.1	46.8
	Last received visit ^g^ (median)	5.0	5.0				
Living conditions	Having Telephone	78.3	94.2		Possibility of using a car		
	Having washing machine	50.0	81.6		Never	75.7	53.5
	Having a fridge at home	96.1	99.7		Having a freezer at home	23.7	56.8

^a^ Social class: **Manual workers**: lower-level employee with administrative or clerical occupation, workers in agriculture, forestry, and commercial fishing, skilled or unskilled manual workers, and housewives. **Non-manual workers**: upper other employers (upper and lower level), own account workers as the lower or upper level, senior officials and upper management, and upper-level employees and family members as an assistant.

^b^ ADL (activities of daily living): five activities as getting in and out of bed, washing and bathing oneself, using the lavatory, dressing, and undressing, and feeding oneself.

^c^Functioning mobility activities: able to move outdoors, walking between rooms, using stairs, walking at least 400 m, and carrying a heavy bag of 5 kg for 100 m.

^d^ Demanding functioning activities: able to cut toenails, cooking, light housework, and heavy housework.

^e^ Social activities: number of participations in social activities: (1) family ceremonies, parties, …, (2) theater, movie, …, (3) visits to clubs, …, (4) library, (5) sports competition watching or participating, (6) religious service, (7) traveling in foreign countries, or (8) traveling in the home country.

All the diseases were categorized as 0: no and 1: yes.

^f^ About a week ago.

^g^ Some days ago.

**Fig. 1:**
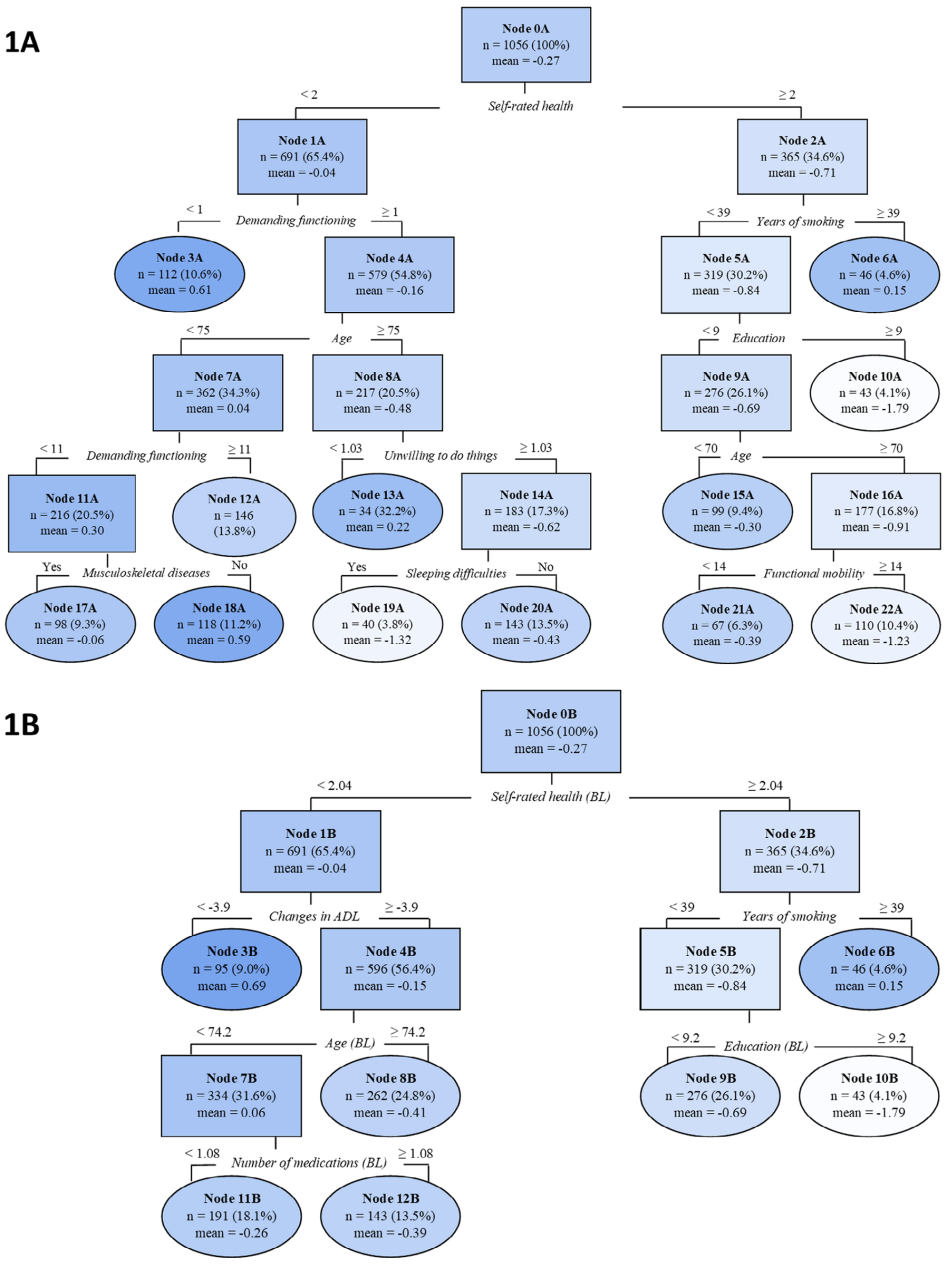
Regression trees for relative measure of longevity (LRPD) fitted on TamELSA data for total population in 1979 with predictors at baseline (1 **A**) and changes for the characteristics over the years added to potential predictors at baseline (1**B**) Notes: Negative values for LRPD indicate a longer life. BL refers to the baseline measurement. A higher score indicates a good SRH, better demanding functioning, and better functional mobility and willingness to do things. Darker shades indicate shorter and lighter shades longer life compared to age and sex peers. Oval shapes represent the terminal nodes i.e., Node 3

Figure 1A and B display the regression trees from the algorithm designed to identify longevity predictors. Figure 1A shows the tree with baseline predictors. The results reveal that SRH was the primary differentiating factor. Those having poor SRH were assigned to the left branch of the tree, and those having an average or good SRH were assigned to the right. Subsequently, several more variables and cut-off points were identified before the algorithm ended in the terminal nodes, i.e., nodes for which no further splits of the nodes would improve the prediction.

The terminal Nodes (oval shape) 10 A (mean LRPD = -1.79, *n* = 43) and 19 A (mean LRPD = -1.32, *n* = 40) had the lowest LRPDs indicating a *longer life than their age and sex peers.* Individuals assigned to Node 19 A differed from those in Node 10 A from the parental node by having poor SRH. Among this group of 75 year or older individuals, individuals lived longer than their peers if they scored at least 1 in demanding functioning, were willing to do things, and had sleeping difficulties. Individuals in terminal Node 10 A had average or good SRH, no or a shorter smoking history than 39 years (the mean was 30 years among smokers), and had nine or more years of education. In addition to these two groups, individuals assigned to Node 22 A (mean LRPD = -1.23, *n* = 122) had a *longer life than their age and sex peers.* They shared the same criteria in the beginning of the tree with the Node 10 A, but had less than nine years of education, were 70 years or older, and had very good mobility (≥ 14).

Those assigned to terminal Nodes 3 A (mean LRPD = 0.61, *n* = 112) and 18 A (mean LRPD = 0.59, *n* = 118) had the highest LRPDs indicating a *shorter life than their age and sex peers* (darkest color). Individuals assigned to Node 3 A were characterized by poor SRH (score < 2) and severe difficulties in performing demanding functioning (score < 1). For those assigned to Node 18 A, demanding functioning appeared two times in the regression tree with different cut-off points (range 0−12). Individuals assigned to Node 18 A, scored ≥ 1 in demanding functioning, were younger than 75 years old, and scored < 11 in demanding functioning at the second time of measurement. Furthermore, they did not have any musculoskeletal diseases.

In the 10-fold cross-validation assessment of the model, the ASE was 2.56 for the model with only the baseline predictors, and it improved very little (2.54) when we added the predictors with changes to the model.

Figure 1B presents the result when changes in longevity predictors were taken into account in addition to the baseline predictors. Terminal Node 3B (mean LRPD = 0.69, *n* = 95) and 6B (mean LRPD = 0.15, *n* = 46) with positive LRPD include individuals with a *shorter life than their peers*. Individuals assigned to Node 3B had poor SRH at baseline and experienced increasing ADL disability (<-4) between 1979 and 1989. Terminal Node 6B that indicated *shorter life than their peers* and Terminal Node 10B (mean LRPD = -1.79, *n* = 43) that indicated *longer life than their peers* remained the same after adding changes in the variables to the regression tree.

The regression trees from the algorithm (Fig. 1A and B) were rather similar with and without changes in the longevity predictors. The number of branches remained the same when changes in the predictors were added to the model, but the number of leaves reduced from 12 to 7. In addition, in the left branch, demanding functioning was replaced with increasing ADL disability, and when splitting predictors for individuals at Node 7B (mean LRPD = 0.06, *n* = 334), demanding functioning was replaced with the number of medications. The age threshold reduced from 75 to 74 and the branch did not continue after Node 8B. The importance of the predictors in the two regression trees for LRPD is presented in Table 2.

**Table 2:** Importance of the predictors for relative measure of the longevity (LRPD) fitted on TamELSA data for total population in 1979 with predictors at baseline (Fig. 1A), and changes in the characteristics over the years added to predictors at baseline (Fig. 1B)

Importance of predictors and count for relative measure of the longevity (LRPD)					
At the baseline 1 A			Changes over the years + baseline (BL) 1B		
Variable	Importance	Count	Variable	Importance	Count
Self-rated health	10.5	1	Self-rated health (BL)	10.5	1
Demanding functioning	9.5	2	Changes in ADL over the years	7.6	1
Age	7.9	2	Years of full-time education (BL)	6.7	1
Years of full-time education	6.7	1	Regular smoking in years (BL)	6.3	1
Regular smoking in years	6.3	1	Number of prescription medicines (BL)	6.0	1
Functional mobility	5.4	1	Age (BL)	5.7	1
Sleeping difficulties	5.0	1			
Musculoskeletal diseases	4.7	1			
Unwilling to do things	4.5	1			

Notes: The importance of the predictor indicates how much a predictor improved the purity of all nodes and can be interpreted as the share of the overall model importance. Importance is scaled to 100. Counts show how many times that predictor appeared in the regression tree

Changing the default values of leaf size, tree depth, or minimum variance did not change the resulting tree substantially. Chronic ischemic heart diseases replaced musculoskeletal diseases in Node 17 A and 18 A in Fig. 1A. Additionally, the variable related to sleeping difficulties did not appear in the sensitivity analysis.

## Discussion

We employed a novel individual-based regression tree analysis to identify predictors of longevity. The used method helps to overcome challenges related to heterogeneity identified in earlier studies, and provides more nuanced insights into predictors of longevity. Our findings suggest that individuals share certain characteristics that lead to a short or long life, but also show the diversity in characteristics leading to distinct survival profiles.

Key predictors of longevity appearing in the regression tree included SRH, demanding functioning, years of education, and smoking history. Other identified predictors were the decline in ADL, number of medications, sleeping difficulties, and unwillingness to do things. The interlinkage of these characteristics and their combined impact on longevity was identified with different pathways.

The study identified three pathways for long life: (1) individuals characterized by good SRH, no or shorter smoking history, and higher education (Node 10), (2) individuals with good SRH, no or shorter smoking history, lower education, and excellent mobility (Node 22), and (3) individuals with poor SRH but able to perform less demanding functions, aged 75 or older, willing to do things, and experiencing sleeping difficulties (Node 19). We also found pathways to shorter life, which were related to poor SRH and difficulties in performing demanding functioning. These pathways demonstrate that individuals may share one or more characteristics leading to longevity, but they can also vary in some criteria and still live long lives. On the other hand, the lack of commonly known predictors for longevity does not guarantee a long life.

Several characteristics underwent change between 1979 and 1989, leading to small alterations in the regression tree when analyzed alongside the baseline characteristics. The number of leaves reduced and two new characteristics emerged; an increase in ADL disability over the years and the number of medications. Our results show a similar predictive value for both the model with only baseline characteristics, and the model with baseline characteristics and changes in the characteristics. ADL disability was the only variable that exhibited changes between 1979 and 1989 and appeared in the regression tree. Thus, the regression trees were mainly based on baseline characteristics. The reasons for this may stem from the fact that due to high mortality, there were only 432 participants with two measurement points. It is also possible that the changes in the variables presenting subjective characteristics were not optimally captured because there were only a few categories in the variables, and the change from one category to another would have required considerable change, for instance, in life satisfaction. Nevertheless, there is research that supports our finding that in a very long follow-up, baseline characteristics may be good predictors of mortality [24].

SRH was the most important predictor of mortality. The association between SRH and mortality is well-established [11, 25]. SRH is an inclusive, dynamic evaluation of general health status [26], which influences behaviors and reflects resources [27] or vice versa [25]. Lorem et al. (2020) found that SRH is a stable predictor of mortality over time, but the association may be weaker if SRH is initially scored as poor [28]. Vogelsang (2014) revealed higher mortality among the oldest old who reported improved SRH compared to those with stable SRH [24]. The study further indicated that individuals with improvements in SRH had worse SRH at the baseline which lagged a greater risk of death [24]. In our study with two measurement points, only baseline SRH predicted mortality, and about 14% of the participants had an improvement in SRH over the years.

Prior research shows that functional disability is a strong predictor of mortality and a shorter life expectancy [29, 30]. For instance, Keeler et al. (2010) reported shortened life expectancy for individuals with disabilities in ADL [31]. In line with the current study, they emphasized the significance of baseline functional status in predicting mortality. ADL is a measure of self-care [31]. We found that in the prediction model with baseline characteristics and changes in the characteristics, a change in ADL was the strongest predictor of mortality after SRH. Disability progresses with difficulty in old age, and the hierarchical ordering of loss of functioning starts from complicated tasks such as cutting toenails and ends in tasks related to self-care [32]. According to Stineman et al. (2012), changes in self-care can predict short and long-term mortality, but the prediction is better for shorter periods [33]. The appearance of ADL in the regression tree instead of demanding functioning after the second measurement point may reflect the fact that the study participants were older, and thus closer to death.

Two earlier identified predictors of mortality, years of education [1] and history of smoking [34], were also found to be among the important predictors in this study. Education is linked to longevity through various mechanisms, and can correlate to higher levels of income, healthier lifestyle, and more stable and better-paid jobs for instance [35]. This study confirmed that a history of smoking is an important predictor of mortality, but we also showed the interlinkage between the years of smoking, years of education, and longevity. Terminal Node 10 with the highest negative LRPD indicates that individuals with an average or good SRH, no or shorter smoking history, and more education lived a longer life than their age and sex peers.

LRPD was built based on an individuals’ age, therefore, the appearance of age in the regression tree algorithm for LRPD was unexpected. Deeg et al. (2018) explained a similar finding as the result of the way of imputing the RPD for individuals alive at the end of the follow-up time [19]. The association of age with the LRPD may indicate a bias towards a healthier selection of the initial study sample. The mean of the RPD (0.47) compared to the theoretical mean of RPD (0.50) indicates that our study sample was slightly healthier / lived longer than the general Finnish population. Therefore, age was considered in the analysis among other predictors.

In general, older age is associated with higher mortality. Our study shows, however, that older age (< 70 / 70+) is one characteristic for longer life in the presence of excellent functioning. This finding highlights the heterogeneity within older individuals, and the need to examine the impact of several predictors of longevity simultaneously.

In addition to the well-established predictors of mortality, we identified four more predictors in the regression trees with less impact on the prediction model. First, the number of medications appeared in the regression tree when baseline characteristics and changes in the characteristics were in the same prediction model. The number of medications or polypharmacy is known to be associated with mortality and morbidity [36, 37]. For instance, Chang et al. (2020) reported that every increase in the number of medications is associated with a 3% increase in mortality [37]. Second, musculoskeletal diseases were present in the prediction model with only baseline characteristics. Musculoskeletal diseases are common in old age [38], but their impact on longevity is controversial. There is, however, evidence from prior research that musculoskeletal diseases increase the risk of mortality [38, 39]. In the sensitivity analysis, this predictor was replaced with chronic heart diseases, which are one of the known predictors of mortality [40]. Third, an unwillingness to do things was identified as a predictor of mortality. Unwillingness to do things or a lack of energy have not been reported as mortality predictors as such before, but they can represent various underlying physical illnesses or psychological disorders [41]. Fourth, we found that having sleeping difficulties was one of the characteristics among those who lived longer than their age and sex peers (Node 19). Previous studies have reported a U-shaped or inconclusive association between sleeping difficulties and mortality [42, 43]. Therefore, our finding highlights the need for further research in this area.

## Strengths and limitations

This study had several strengths. We used novel approaches which enabled the use of an individual-based measure of longevity, and applied the advanced method of algorithmic regression tree analysis. This approach allowed us to simultaneously consider multiple potential predictors of longevity while accounting for interactions between them, even when predictors are mutually related. This allows us to reveal the most important combinations leading to longevity. Unlike traditional models which might suffer from omitted variable bias when key predictors are excluded, the regression tree approach identifies the most important combinations of predictors without such limitations. The study had a long follow-up of 35 years. As most of the participants had died during the follow-up period, we used the exact dates of death to calculate the RPD. In addition, two measurement points enabled the examination of how changes in the predictors over ten years affected the prediction of longevity.

However, we acknowledge that the study also has some limitations. First, algorithmic regression trees are easily interpretable, but suffer from instability from one analysis to another. This instability can represent the effect of a correlation, and while the tree may look different, the same underlying features are still there. That said, altering the default values of leaf size, tree depth, or minimum variance (as a sensitivity analysis) did not substantially change the resulting tree in our study. As a second consideration, we had only two measurement points ten years apart. Especially in the older population, health can deteriorate rapidly. Thus, the ten year interval is not optimal to capture the changes that take place. Furthermore, due to the participants’ mortality, the second measurement point had a smaller study population, which may have had an impact on the finding that the regression tree was mainly based on baseline characteristics.

## Conclusions

The novel approach used in this study identified the same key predictors of longevity as more traditional approaches: SRH, functioning, years of education, and history of smoking. The simultaneous examination of a broad range of potential predictors revealed that longevity can be achieved under diverse conditions, and that heterogeneous groups of people achieve it. In this study concerning an older population with a 35 years of mortality follow-up, the examined changes in longevity predictors over time did not change the main results.

## Electronic supplementary material

Below is the link to the electronic supplementary material.

Supplementary Material 1

## Data Availability

The datasets used and analyzed during the current study are available from corresponding author on a reasonable request.

## Declarations

The Tampere Longitudinal Study on Aging (TamELSA) started as part of the World Health Organization’s (WHO) Eleven Country Study on Health Care in 1979. In the TamELSA study, the data collection is historically old. At the time of data collection, data was collected only by interviews and no hospital record data was used. The researchers also did not need an ethical statement as there was no specific Finnish legislation governing non-medical studies in place at that time.

At that time, the participants were sent a letter in which the aims and procedures of the study were explained, and a time for a face-to-face interview was suggested. Voluntary participation was emphasized, and if the participant agreed to meet the interviewer and participated in the interview, this was regarded as implicit consent to participate in the study.

However, later, authorizations to link with national mortality registers were obtained from the national register authority, Statistics Finland, and the Population Register.
