# Supplementary material for: Tree-based analysis of longevity predictors and their ten-year changes: a 35-Year mortality follow-up
Source: BMC Geriatr. 2024 Oct 11;24:817. doi: 10.1186/s12877-024-05404-4 (PMC11468105; doi:10.1186/s12877-024-05404-4)
Supplement: Supplementary file 1 — Supplementary Material 1 [file 12877_2024_5404_MOESM1_ESM.docx]

**Appendix**

Table: Description of the variables in the article “Tree-Based Analysis of Longevity Predictors and Their Ten-Year Changes: A 35-Year Mortality Follow-Up”. Highlighted variables were subjected to changes between 1979 and 1989.

| **Variable/ question** | **Response** |
| --- | --- |
| **Sex** | 1) male, 2) female |
| **Social class** | Non manual:  Senior officials, upper management and upper-level employees, farmers on their own account  Manual:  Skilled and unskilled workers, farmers, workers in agriculture, forestry, commercial fishing, lower-level employers, and housewives were distinguished as manual workers. |
| **Marital status** | 1) never married, 2) married, 3) widowed, 4) divorced, 5) separated, and 6) others  scored as 1) never married, 2) married, 3) widowed, and 4) divorced, separated and others |
| **Years of full-time education** | 0−25 |
| **Activities of daily living (ADL)** was measured by the five activities:  Getting in and out of bed  Washing and bathing oneself  Using the lavatory  Dressing and undressing  Feeding oneself | 1) can do without difficulties, 2) can do with difficulties but without help, 3) can do only with help, and 4) cannot do.  Scored as 0) cannot do, 1) can do only with help, 2) can do with difficulties but without help, and 3) can do without difficulties.  ADL was scored between 0−15. It is a sum of scores over the five variables. |
| **Mobility** was measured by:  Moving outdoors  Walking between rooms  Using stairs  Walking at least 400 m,  Carrying a heavy bag of 5 kg for 100 m | 1) can do without difficulties, 2) can do with difficulties but without help, 3) can do only with help, and 4) cannot do.  Scored as  0) cannot do, 1) can do only with help, 2) can do with difficulties but without help, and 3) can do without difficulties.  Mobility was scored between 0−15. It is a sum of scores over the five variables. |
| **Demanding functioning** was measured by:  Cutting toenails  Cooking  Light housework  Heavy housework | 1) can do without difficulties, 2) can do with difficulties but without help, 3) can do only with help, and 4) cannot do.  Scored as 0) cannot do, 1) can do only with help, 2) can do with difficulties but without help, and 3) can do without difficulties.  Demanding functioning was scored between 0−12. It is a sum of scores over the four variables. |
| **Do you have any diseases which affect ADL?**  Diseases according to the Finnish edition of the international classification of diseases (ICD-9) 1975,  Infection, neoplasm, endocrine diseases, Diseases of blood, mental disorder, Nervous system diseases, respiratory, digestive, genito-urinary, musculoskeletal, cardiovascular disease was divided into three categories: hypertension, ischemic heart diseases, and other cardiovascular diseases (circulatory) | 0) no, 1) yes |
| **Doing physical exercise** | yes or no |
| **Number of years smoking regularly** |  |
| **Self-rated health**  How you evaluate your present health? | 1) very good, 2) fairly good, 3) average, 4) fairly bad, 5) bad  scored as 0) bad, 1) fairly bad, 2) average, 3) fairly good, 4) very good. |
| **Social activities** were measured by the number of occasions of participation in social activities during past 12 months as  a) family ceremonies, parties, weddings, funerals  b) theaters, movies, concerts, art exhibitions  c) visits to clubs or societies  d) library  e) sport competitions watching or taking part  f) religious services,  g) traveling foreign countries or home country | It was scored between 0-51. |
| **Last visit received**  When did somebody last visit you?  **Last visit paid**  When did you last visit someone? | 1) today or yesterday, 2) some days ago, 3) about a week ago, 4) about two weeks ago, 5) about a month ago, 6) about half years ago, or 7) more than half a year ago.  scored as 0) more than half a year ago, 1) about half years ago, 2) about a month ago, 3) about two weeks ago, 4) about a week ago, 5) some days ago, 6) today or yesterday |
| **Being alone**  How often are you alone? | a) often, b) rarely, c) never  scored as 0) often, 1) rarely, 2) never |
| **Assisting in bringing up grandchildren** | 1) No, 2) Yes |
| **Having good friends** | 1) No, 2) Yes |
| **Feeling forgotten**  Do you feel forgotten? | 1) often, 2) sometimes, 3) never, 4) cannot say  scored as 0) often, 1) sometimes and cannot say, 2) never |
| **Feeling unnecessary**  Do you feel unnecessary?  For the coding of the cannot say response we used the Kaplan-Meier analysis and combined this category with the closest curve to it. | 1) often, 2) sometimes, 3) never, 4) cannot say  scored as 0) often, 1) sometimes and cannot say, 2) never |
| **Satisfaction with his/her economic situation**  How do you feel about your economic situation? | 1) good, 2) satisfactory, 3) bad, 4) cannot say  scored as 0) bad and cannot say, 1) satisfactory, 2) good |
| **Satisfaction with human relationship**  Are you satisfied with the human relationships which you have had or made during the course of your life? | 1) satisfied, 2) unsatisfied, 3) cannot say |
| **Feeling tired of life**  Do you feel tired of life?  For the coding of the cannot say response we used the Kaplan-Meier analysis and combined this category with the closest curve to it. | 1) often, 2) sometimes, 3) never, and 4) cannot say  scored as 0) often, 1) sometimes and cannot say, 2) never |
| **Feeling lonely**  Do you feel lonely?  For the coding of the cannot say response we used the Kaplan-Meier analysis and combined this category with the closest curve to it. | 1) often, 2) sometimes, 3) never, and 4) cannot say  scored as 0) often and cannot say, 1) sometimes, 2) never |
| **Satisfaction with life**  How satisfied are you with present life?  For the coding of the cannot say response we used the Kaplan-Meier analysis and combined this category with the closest curve to it. | 1) very satisfied, 2) satisfied, 3) reasonably satisfied, 4) unsatisfied, 5) very unsatisfied, or 6) cannot say.  scored as 0) cannot say and very unsatisfied, 1) unsatisfied, 2) reasonably satisfied, 3) satisfied, 4) very satisfied |
| In the last two weeks have you suffered from:  **Tiredness or feeling of faintness**  **Unwillingness to do things or lack of energy**  **Worsening of memory**  **Low spirits or depression**  **Pain in the joints**  **Significant changes in weight**  **Difficulties in falling asleep** | 1) no, 2) yes, occasionally, 3) yes, often, and 4) yes, nearly continuously  scored as 0) yes, nearly continuously, 1) yes, often, 2) yes, occasionally, 3) no |
| **Number of medications** prescribed by a doctor |  |
| **Hearing problem** was the combination of three questions:  using a hearing aid with yes or no options,  hearing in a face-to-face situation,  hearing in a group | 1) yes, 2) no  1) yes, 2) no  1) yes 2) no and 3) yes with difficulty  If the answers for these three questions were yes it was categorized as yes (0), and the rest was categorized as no (1). |
| **Having a washing machine** | 0) no, 1) yes |
| **Having a telephone** | 0) no, 1) yes |
| **Having a freezer** | 0) no, 1) yes |
| **Having a refrigerator** | 0) no, 1) yes |
| **Possibility of using a car**  Do you have a use of a car? | 1) never, 2) sometimes, 3) always  scored as 0) never, 1) sometimes and always |
